# Supplementary material for: Vulnerability to memory decline in aging revealed by a mega-analysis of structural brain change
Source: Nat Commun. 2025 Nov 21;16:11488. doi: 10.1038/s41467-025-66354-y (PMC12749543; doi:10.1038/s41467-025-66354-y)
Supplement: Supplementary file 1 — Supplementary Information [file 41467_2025_66354_MOESM1_ESM.pdf]

Supplementary information to “Vulnerability to memory decline in aging revealed by a mega-analysis of structural brain change”

Methods

Participants

The datasets include the LCBC[1], Betula[2], UB[3,4], and BASE-II[5,6] datasets (from the Lifebrain Consortium)[7] as well as the COGNORM[8], the Alzheimer’s Disease Neuroimaging Initiative (ADNI) database (https://adni.loni.usc.edu)[9], AIBL[10], BBHI[11], the Harvard Aging Brain Study (HABS)[12], the UKB (https://www.ukbiobank.ac.uk/)[13], PREVENT-AD[14,15], OASIS3[16], and VETSA[17] datasets. Based on preprocessing requirements, MRI data from scanners with fewer than 25 observations were also excluded based on stability issues in the normative modeling-based harmonization. For computing slopes, individuals with less than 1.5 years of follow-up, either of memory function or brain structure, were also excluded. For memory–brain pairing, individuals without at least partially overlapping follow-up intervals for brain structure and memory were excluded, as well as those in which one of the assessments (in practice, memory function only) started > 10 years before or ended > 10 years after the beginning or end of the MRI follow-up period. The initial dataset included individuals with 1 to 14 MRI acquisitions with longitudinal structural MRI data spanning up to 15.8 years. Similarly, memory assessments range from 1 to 30 observations per individual with a follow-up up to 31.6 years.

ADNI: The Alzheimer’s Disease Neuroimaging Initiative (ADNI)[9] is a multi-site project led by Dr. Michael W. Weiner to assess the progression of mild cognitive impairment (MCI) and early Alzheimer’s Disease (AD), combining imaging, clinical and other biological markers, and neuropsychological and clinical assessments over time. For more information, visit https://adni.loni.usc.edu/about/. The age range for the participants is 55-90 years. The present study includes participants from ADNI 1, ADNIGO, ADNI2, and ADNI 3, who were cognitively healthy at baseline (*DX_bl* variable). Only observations in which participants were

still cognitively healthy were included as determined by the ADNI team (*DX* variable). Amongst others, participants were required to have no evidence of ischemic stroke (Hachinski Ischemic Score ≤ 4), a Geriatric Depression scale score < 6, stable medications for 4 weeks before the screening, good auditory and visual acuity, good general health, no medical contraindications to MRI, and at least 6 grades of education/work history. In-depth general inclusion and exclusion criteria are described elsewhere[18]. All participants signed an informed consent form, and the protocols were approved by the corresponding regional ethical committees in the US and Canada. Data was retrieved in April 2021.

AIBL: The Australian Imaging, Biomarker & Lifestyle Flagship Study of Ageing (AIBL)[10] is a prospective study including cognitively normal participants, and patients with mild cognitive impairment, or AD aged 60 years or older. The study assesses the biomarkers, genetic factors, cognitive characteristics, and health and lifestyle factors that are associated with the development of AD, combining techniques such as MRI, positron emission tomography (PET), blood tests, and fluid sample analysis, as well as neuropsychological and clinical assessments. Healthy participants must meet specific criteria, including being free of cognitive impairments and having test performance within 1.5 standard deviation *(SD)* of age-adjusted norms. The test battery and sample description, as well as in-detail general inclusion criteria, have been described in detail previously[10]. All observations corresponding to cognitively normal individuals were included, as defined and evaluated by the AIBL team. All participants signed an informed consent form and the protocol was approved by the institutional human research committees of Austin Health, St Vincent's Health, Hollywood Private Hospital, and Edith Cowan University (Australia). Data was retrieved in February 2023.

BASE-II: Participants of the Berlin Aging Study II (BASE-II) were community-dwelling older adults recruited from the greater Berlin metropolitan area through advertisements in newspapers and public areas[5,6]. The baseline sample comprised 2200 participants: 1600 older adults aged 61–88 years, and 600 younger adults aged 24–40 years. Participants were invited to a medical exam and cognitive testing sessions. After completion of the cognitive examination of BASE-II, eligible participants were invited to take part in one MRI session within a time window of 2–4 weeks after cognitive testing. The MRI sample consisted of 341 older adults aged 61–82 years and 103 younger adults. MR scans and cognitive scores were obtained in 2012-2013. A subsample of the MR sample was later re-invited for follow-up. The different

elements of the study were approved by the ethics committees of the Max Planck Institute for Human Development, the Charité University ethics committee, and by the ethics committees of the German Association for Psychology (DGPs). Participants signed written informed consent and received monetary compensation for their participation in BASE-II and the MRI study. All experiments were performed in accordance with relevant guidelines and regulations. Inclusion criteria for taking part in this study were age between 20 and 35 or 60 and 80 years, apparently healthy. Exclusion criteria were untreated diabetes and hypertension; prior stroke, head injuries, or brain surgery; psychiatric illness; major depression; dementia with a score < 24 on the Mini- Mental State Examination. None of the participants took medication that might affect memory function or had a history of head injuries, medical (e.g., heart attack), neurological (e.g., epilepsy), or psychiatric disorders (e.g., depression). The BASE-II cohort was part of the Lifebrain obtained as part of the Lifebrain consortium[7]. All participants reported normal or corrected to normal vision. Observations with MMSE < 26 or no MMSE data were discarded. Data was retrieved in February 2022.

BBHI: Barcelona Brain Health Initiative study (BBHI; https://bbhi.cat/en/) participants are community-dwelling individuals between 40 and 65 years of age, of both sexes, free from any self-reported neurological or psychiatric diagnosis at the time of recruitment[11,19]. It is an ongoing longitudinal cohort study that aims to investigate the determinants of brain and mental health in healthy middle-aged and older adults. Recruitment started in 2017, when multiple initiatives (including conferences, radio and television interviews, and social media advertisements) took place to encourage participants to join the study. It has enrolled 4,686 participants via a web-based application, who completed a first online questionnaire. The BBHI includes periodic cognitive, medical, brain imaging, and biological assessments. BBHI has several sub-studies. In one of them, a sample of 1,000 participants is undergoing a detailed clinical phenotyping through a multi-day in-person evaluation that includes cognitive, physical, and medical assessments, biological sample recollection, structural and functional magnetic resonance imaging (MRI), and electroencephalography (EEG). Participants of this study are invited biannually for repeated evaluations. Data was retrieved in July 2023.

BETULA: The BETULA project (Umeå)[20,21] is a prospective longitudinal study on aging, memory, and dementia, which used a population-based sampling of healthy middle-aged and older adults for recruitment. Detailed recruitment procedures are found elsewhere[20,21]. For the

current analyses, the MRI subsample of the study is used. Participation in the neuroimaging study was offered to all participants who had remained in the study and completed cognitive testing at the 5th Betula test wave onwards. Exclusion criteria were severe visual or auditory handicaps, intellectual or developmental disabilities, suspected dementia, having a mother tongue other than Swedish, MRI contraindications, severe neurological disorders, or visual/motor deficits that could interfere with fMRI data collection, MMSE <24, brain or head surgery, and substantial brain anatomical deviations. Some participants were later excluded due to discovered neurological conditions, severe depression, and MRI anatomical abnormalities. All participants signed an informed consent, and the protocols were approved by the Regional Ethical Vetting Board at Umeå University. The BETULA cohort was part of the Lifebrain obtained as part of the Lifebrain consortium[7]. Data was retrieved in August 2022.

COGNORM: The COGNORM cohort[22] is an ongoing, prospective study coordinated by the Oslo University Hospital and Diakonhjemmet Hospital, Oslo, Norway. Patients (age ≥ 65 years) scheduled for elective gynecological, urological, or orthopedic surgery under spinal anesthesia were recruited. Participants were required to have no dementia, previous stroke with sequela, Parkinson's disease, or other neurodegenerative diseases that are likely to affect cognition. Patients with suspected undiagnosed dementia at any time within the first five years of follow-up (n = 15)[23], MMSE score <28 at baseline, and at least two abnormal cognitive test scores (-1.5 *SD* below the mean normal value for age, sex, and education) were excluded. All observations corresponding to cognitively normal individuals were included. All participants signed an informed consent form, and the protocol was approved by the Norwegian Regional Committees for Medical and Health Research Ethics and the Data Protector Officer at Oslo University Hospital. Data was retrieved in March 2023.

HABS: The Harvard Aging Brain Study (HABS)[24] is an ongoing, long-term observational study that aims to enhance our understanding of brain aging and the early stages of Alzheimer's disease. The study collects PET, MRI data, neuropsychological, and clinical assessments. The age range was between 50 and 90 years at the time of baseline assessment, and all patients were considered non-clinically impaired at the start of the study. Further participants had a CDR score of 0, MMSE score ≥ 25, < 11 on the Geriatric Depression Scale, and scores above age- and education-adjusted cutoffs on the 30-Minute Delayed Recall of the Logical Memory Story A to be included in the study. Participants with a history of alcoholism, drug abuse, head

trauma, or current serious medical/psychiatric illness were excluded. Further details can be found elsewhere[24]. Observations with MCI or AD diagnostic (DX variable) were excluded. All participants signed an informed consent form, and the protocol was approved by the Partners Healthcare Human Research Committee. Data consisted of the "HABS data release 2.20", retrieved in August 2022 via habs.mgh.harvard.edu.

LCBC: The Center for Lifespan Changes in Brain and Cognition cohort (LCBC, Oslo)[1,25] consists of cognitively healthy, community-dwelling participants across the lifespan and is drawn from studies coordinated by the LCBC Research Group (LCBC www.oslobrains.no), approved by a Norwegian Regional Committee for Medical and Health Research Ethics. Written informed consent was obtained from all participants. The samples were recruited by a variety of methods, such as newspapers and webpage ads. Most participants were recruited for observational studies, some currently ongoing, while a minority were recruited to enter into cognitive training. Written informed consent was obtained from all adult participants. All participants had to undergo a standardized health interview before being included in the study, and those with a history of neurological or psychiatric conditions or who reported concerns about their cognitive function were excluded. Additionally, all participants over the age of 40 years were required to score at least 25 on the Mini-Mental State Examination. The LCBC cohort was part of the Lifebrain obtained as part of the Lifebrain consortium[7]. MRI observations paired with MMSE ≤ 25 were excluded. Data was retrieved in November 2022.

OASIS3: The Open Access Series of Imaging Studies (OASIS3)[26] is a retrospective collection of multimodal data that focuses on aging and AD and is openly accessible to the scientific community. OASIS-3 includes neuroimaging, clinical and neuropsychological data. Participants were recruited through the Washington University Knight Alzheimer Disease Research Center via flyers, word of mouth, and community engagements, and were aged between 42 and 95 years. Only participants deemed cognitively normal at baseline were included in the observations. Exclusion criteria included medical conditions that precluded longitudinal participation or medical contraindications for the different study arms. See in-detail inclusion and exclusion criteria[26]. All participants consented to Knight ADRC-related projects following procedures approved by the Institutional Review Board of Washington University School of Medicine. Observations were included until the last observation in which a subject was

deemed cognitively healthy as determined by the Clinical Dementia Rating Scale (CDR)[27]. Data was retrieved in November 2022.

PreventAD: The Pre-symptomatic Evaluation of Experimental or Novel Treatments for AD (PREVENT-AD)[28] is a retrospective, long-term study that follows cognitively healthy older individuals with a familiar history of AD. It includes participants enrolled either from an observational cohort or the clinical trial of PREVENT-AD. This study comprises MRI images, blood and CSF samples, and clinical and neuropsychological assessments. Participants in the study had to be at least 60 years old, had ≥6 years of education, and they needed to be cognitively unimpaired at baseline. The Montreal Cognitive Assessment (MoCA) and CDR scales were used to assess cognitive abilities, and participants were considered cognitively intact if their MoCA scores were ≥ 26/30 or their CDR was = 0. Other exclusion criteria at baseline included medical conditions that prevented longitudinal participation or medical contraindications to MRI, use of acetylcholinesterase inhibitors, other approved prescription cognitive enhancers, hypertension, or substance abuse. The inclusion and exclusion criteria have been previously described in detail[28]. The protocols, consent forms, and study procedures were approved by the McGill Institutional Review Board and the Douglas Mental Health University Institute Research Ethics Board. Observations with RBANS > 1 *SD* below the mean and probable MCI, as evaluated by a clinician, were excluded. Data was retrieved in February 2022.

UB: The University of Barcelona cohort consisted of a series of retrospective sub-studies studies[3,4,29,30]. In all cases, samples consisted of cognitively healthy, community-dwelling participants with normal cognitive and visual function. Most participants were recruited for observational studies, while a minority were recruited to enter into cognitive training. Exclusion criteria varied a bit across studies (see specific studies for details), but include severe neurologic and psychiatric disorders, recent head trauma or brain surgery, cognitive deterioration, or dementia with a score < 24 on the Mini-Mental State Examination and additional neuropsychological criteria, other neurodegenerative disorders like Parkinson's disease and chronic illness with a projected shortened lifespan. Follow-up observations with MMSE < 26 were excluded. All participants signed an informed consent, and the protocols were approved by the ethical committees of the University of Barcelona and of the Hospital

Clinic of Barcelona. The UB cohort was part of the Lifebrain obtained as part of the Lifebrain consortium[7]. Data was retrieved in February 2022.

UKB: The UK Biobank (UKB) (https://www.ukbiobank.ac.uk/about-biobank-uk/) is a major national and international health resource with the aim of improving the prevention, diagnosis, and treatment of a wide range of illnesses. UK Biobank recruited ≈500,000 people aged between 40-69 years in 2006-2010 from across the country to take part in this project[31]. Potential participants were identified through National Health Service (NHS) registers according to being aged 40-69 and living within a reasonable traveling distance of an assessment center. Assessment centers are located in accessible and convenient locations with a large surrounding population. Participants have undergone measures, provided samples, and detailed information about themselves, and agreed to have their health followed. The study sample was drawn from the UK Biobank neuroimaging branch[13] and conducted under data application number 32048. Only individuals with longitudinal MRI data were used in this study. Note that the MRI showcase data was not used. Rather, MRI data was re-processed inhouse with the longitudinal pipeline. Participants signed an informed consent and the protocols were approved by the North West Multi-Center Research Ethics Committee [MREC]; see also https://www.ukbiobank.ac.uk/the-ethics-and-governance-council.

VETSA: The Vietnam Era Twin Study of Aging (VETSA) is an ongoing large-scale investigation of cognitive aging and brain aging in men[17,32,33]. It primarily investigates genetic and environmental influences on cognitive aging, brain structure and function, and health. VETSA involves over 1600 male twins from the Vietnam Era Twin Registry who served during the Vietnam War era. Assessments began when participants were in their 50s (in 2003) and follow-ups are conducted every 5-6 years. The age range of participants is narrow (from 52 to 60 at baseline). It has, amongst other things, extensive neurocognitive testing, legacy cognitive data, genome, MRI, and plasma biodata. Over 1200 twins participated in waves 1, 2, and 3. All VETSA participants are men and all were in some branch of military service at some time between 1965 and 1975, though approximately 80% report no combat experience. The sample is a reasonably representative, community-dwelling sample of men in their age range in the USA. Attrition-replacement procedures were taken in wave 2. VET registry was created by inviting to participate male twins who served in the United States military during the Vietnam Era by identifying them through their birth year (between 1939–1957), period of active duty

(between 1965–1975), and having the same last name, different first name, same date of birth, and same first five digits of the Social Security number. VETSA twins are recruited from a participant pool (*n* = 3322) of a previous research study (Harvard Twin Study of Drug Abuse and Dependence), which attempted to contact all available VET Registry members (*n* = 7375). Recruitment for the VETSA study involved identifying eligible twins through the VET Registry at the VA Medical Center in Seattle. A survey firm then contacted potential participants with an introductory letter, followed by a phone call. Interested individuals submitted an authorization form indicating consent and site preference. These forms were processed and forwarded to the respective study sites, which then contacted participants. For the VETSA MRI study, participants were screened for safety issues (e.g., MRI contraindications), and both members of a twin pair had to consent to participate. For MRI, twins had to additionally be able to travel to a scanning site. Other exclusion criteria depended on exclusion criteria for serving in the military, e.g., participants scoring in the lowest 10th percentile ranks of the Armed Forces Qualification Test (AFQT) were excluded from the military. In addition, we further excluded data from participants with incidental radiological findings, history of seizure, and diagnosis of multiple sclerosis, and AIDS. Data was retrieved in July 2023.

MRI Acquisition, preprocessing, and harmonization

MRI acquisition and preprocessing. For datasets not provided in Brain Imaging Data Structure (BIDS) format, data was converted to BIDS[34]. BIDS transformation of ADNI, AIBL, OASIS3, and HABS data was performed with Clinica software[35,36]. We used the longitudinal FreeSurfer v.7.1.0 stream[37] for cortical reconstruction and volumetric segmentation of the structural T1w scans[38,39]. For sessions with multiple scans, data from the scanners were averaged. Briefly, the images were processed using the cross-sectional stream, which includes the removal of nonbrain tissues, Talairach transformation, intensity correction, tissue and volumetric segmentation, cortical surface reconstruction, and cortical parcellation. Next, an unbiased within-subject template space based on all cross-sectional images was created for each participant, using robust, inverse-consistent registration[40]. The processing of each time point was then reinitialized with common information from the within-subject template to increase reliability and statistical power. Except for the BETULA dataset, all data was preprocessed on the Colossus processing cluster, part of the Tjenester for Sensitive Data (TSD) (https://www.uio.no/tjenester/it/forskning/sensitiv/), University of Oslo (UiO).

Data harmonization. Brain regions were harmonized using a normative modelling framework[41,42] with the *PCNtoolkit* (v.0.30.post2), in *Python3* environment[43] (v.3.9.5). This framework offers several advantages as it i) is carried independently across sites, ii) can isolate site-effects from other sources of variance associated with it, and iii) produces site-agnostic deviation scores (z-statistics) adjusted for age and sex. *PCNtoolkit* uses a Hierarchical Bayesian Regression (HBR) technique[44] and pretrained models from 82 different datasets, including the UKB. New sites can be added to the model using a held-out calibration dataset. To avoid losing longitudinal observations, here, we performed calibration recursively by iteratively (n = 100) holding out a calibrating sample and computing the estimates on the remaining data. The average scores of all iterations were used as the standardized scores for each observation. Scanners contributing with < 12 unique individuals or < 25 observations were removed. For scanners contributing > 12 and < 32 unique individuals, we used a calibration sample consisting of all but 2 participants and estimated the harmonized scores in these two. For scanners with >= 32 unique individuals, we used, in each iteration, a held-out sample of 30 individuals, while

estimates were applied to the rest. Most scanners with < 32 unique individuals belonged to ADNI due to its multisite nature. Note this step was performed with the initial MRI sample, i.e., regardless availability of longitudinal MRI data or paired memory function assessments. In most regions, normative scores show high reliability across iterations (intraclass correlation across regions [*ICC*] (.97[.01], range = .95 - .97). For longitudinal brain change, the mean reliability was even higher (.98[.01], range = .97 - .98). This is expected, since the uncertainty (error) is shared across observations of the same individual as long as the scanner does not change, i.e., the most recurring scenario. The correlation between harmonization with a normative modelling framework and Generalized Additive Mixed Models (GAMMs) – using age as a smoothing term, sex as a fixed factor, and dataset and scanner as random intercepts was $r = 0.98$ (0.03). The only regions with a correlation $r < 0.90$ were ventricle regions. This is likely due to increased dispersion of ventricular volume with age. Normative modelling models age variations in variability and converts the data to Z-scores based on age and sex, while GAMMs model age changes in the mean but not in the variability. Next, we selected individuals with at least 2 observations and a minimum follow-up of 1.5 years. The follow-up criterion were chosen to exclude data with very low reliability for capturing long-term brain change, i.e., brain aging[45]. For each individual and region, we estimated yearly change by regressing normative MRI values on follow-up time (Δbrain). Brain change data was Z-standardized by each site and coupled with memory change data for higher-level analyses. In practice, Δbrain reflects the yearly brain change of an individual relative to their age and sex peers. For pairing Δbrain with Δmemory, individuals had to have – at least partially - overlapping follow-up intervals, while the non-overlapping period had to be lower than 10 years.

Results

Hemispheric asymmetries in the association between brain change and memory change.

No strong evidence for asymmetry in change - change associations was found (paired t-test; $\Delta\beta_w$*[left-right]* = 0.007, $t(82) = 1.78$, $p = 0.079$) (Supplementary Figure 2a). However, leftward asymmetry in change - change association was found at age 50 ($t(82) = 2.66$, $p_{FDR} = 0.05$). Asymmetry estimates in change - change associations decrease with increasing aging ($\Delta\beta_{w[left-right]}$*[CI]* = 0.017[.001, .033], 0.016[.004, .028], 0.008[-.001, .018], 0.001[-.009, .101], -0.002[-.002, 0.010] at 40, 50, 60, 70, and 80 years of age) (Supplementary Figure 2b).

Education as a resilience or a cognitive reserve factor?

We operationalized education level by classifying individuals as having or not having, tertiary education. In UKB and BBHI, education was reported categorically, based on ISCED level 7 and ISCED level 3 nomenclature, respectively, and thus categories were merged as appropriate. The remaining datasets reported years of education. For the ADNI, BASE-II, BETULA, COGNORM, HABS, LCBC, OASIS3, and VETSA, individuals with ≥ 16 years of education were classified as having tertiary education. For preventAD and BBHI, the threshold was set at ≥ 15 years, reflecting Quebec scholarization norms https://www.quebec.ca/en/education/study-quebec/education-system and the mid-20th-century Spanish education system. The AIBL dataset did not have available information on education. See an in-depth description in Fjell and colleagues[46]. Also, 17 individuals from BASE-II and 19 from LCBC did not have education level. The analyses were carried out with 3559 individuals with longitudinal information. We examined the following effects of education level on brain and memory decline: 1) education level as predictor of memory decline; 2) education level as predictor of regional brain decline; 3) education level × age interaction on memory decline; 4) education level × age interaction on regional brain decline; 5) education level × regional brain decline on memory decline; 6) education level × age × regional brain decline on memory decline. In all models, dataset was included as a random intercept. Analysis 1-2 were conducted using a linear mixed-effects models as implemented in *lme4* (v.1.1-37), *lmerTest* (v.3.1-3)[47,48]. The remaining analyses were performed using *GAMMs* as implemented in the *mgcv* (v.1.9-3) *R-package*[49], with statistical significance assessed via a bootstrapping approximation as described in the main text for all GAMMs.

Neither education level nor the education level × age interaction were associated with memory decline ($\beta[CI]$ = 0.014[-.04,.07], $t(2616)$ = 0.39, p = 0.69; edf = 2.3, p = .96). After correcting for multiple comparisons, both education level and the education level × age interaction were also unrelated to brain decline (p_{FDR} > .3). Likewise, no significant effects were observed for the education level × brain decline nor the education level × brain decline × age on memory decline interactions (p_{FDR} > .3). See complete stats in Supporting app. The results are in line with those

reported in Fjell and colleagues[46] in a largely overlapping sample using instead a main factor of global decline.

Independent regional contributions to memory decline.

We employed GAMMs to investigate whether brain changes within specific clusters (refer to Figure 3, Supplementary Table 3) were associated with memory change. We conducted 4 analyses: i) basic, without additional covariates; ii) controlling for the hippocampus-based cluster; iii) controlling for the main factor (PC1) of brain decline; and iv) including all clusters together in a single model. Dataset was used as a random intercept. Detailed statistics are available in Supplementary Table 4. All clusters showed significant Δbrain – Δmemory associations without adjusting for covariates (all *p*'s ≤ .001). This aligns with expectations, since all regions in the clustering analyses demonstrated associations with memory change. When controlling for the hippocampal cluster (#1), five clusters showed significant Δbrain – Δmemory associations: cluster #3, the left and right amydala (β_w = .11, *edf* = 1.2, *p* = 0.01); cluster #5, left pericallosal sulcus, right straight gyrus, and right subcallosal gyrus (β_w = .16, *edf* = 3.1, *p* = 0.002), cluster #6, right long insular gyrus and planum polare (β_w = .06, *edf* = 1.0, *p* = 0.01); cluster #7, right transverse temporal sulcus (β_w = .05, *edf* = 3.0, *p* = 0.02); and cluster #8, left parahippocampal gyrus (β_w = .05, *edf* = 4.4, *p* = .03). When controlling for a main factor of global decline, four clusters showed significant Δbrain – Δmemory associations: cluster #3, the left and right amydala (β_w = .09, *edf* = 2.8, *p* = 0.05); cluster #5, left pericallosal sulcus, right straight gyrus, and right subcallosal gyrus (β_w = .11, *edf* = 3.0, p = 0.01), cluster #7, right temporal transverse sulcus (β_w = .03, *edf* = 3.1, *p* = 0.01); and cluster #4, right supramarginal gyrus (β_w = -.07, *edf* = 1.0, *p* = .02). The lack of Δbrain – Δmemory associations for the hippocampal cluster is probably explained by the fact that the factor of global decline weights heavily on the left and right hippocampus (Figure 3c). When included in a single model, five clusters showed significant Δbrain – Δmemory associations: cluster #1, the left and right hippocampus and left inferior lateral ventricle (β_w = .09, *edf* = 3.8, *p* = 0.001); cluster #3, the left and right amygdala (β_w = .08, *edf* = 1.0, *p* = 0.03); cluster #5, left pericallosal sulcus, right straight gyrus, and right subcallosal gyrus (β_w = .11, *edf* = 2.6, *p* = 0.01), cluster #7, right temporal transverse sulcus (β_w = .03, *edf* = 2.7, *p* = 0.04); and cluster #8, left parahippocampal gyrus (β_w = .07, *edf* = 4.6, *p* = .02). Note that β_w was estimated on above-average brain decliners, due to the non-linear nature of the associations (Figure 1). Note also that all p-values, except for the single model analysis, were obtained via bootstrapping. For the single model analysis, the p-values were derived from the implementation in the *mgcv R-package*[49]

as bootstrapping proved overly conservative, possibly due to cross-correlations between clusters.

Sensitivity analysis using APOE ε4 non-carriers-only subsample.

To evaluate the potential influence of APOE ε4 on the Δbrain – Δmemory associations, we conducted analyses exclusively within a sample of APOE ε4 non-carriers and compared the results to the overall sample. 99.94% of the estimates in APOE ε4 non-carriers fell within the confidence interval of the estimates from the overall sample. 17 out of 19 regions that were significant in the entire sample were also significant ($p < .05$) in the APOE ε4 non-carrier sample. The exceptions were the left parahippocampal and right supramarginal gyri, where Δbrain – Δmemory associations did not reach significance ($p = 0.05$). Further, the influence of age on the Δbrain - Δmemory association was also consistent within the APOE ε4 non-carrier sample. 92.18% of the estimates from APOE ε4 non-carriers fell within the confidence intervals of the main sample. The left putamen, right parahippocampal gyrus, and left temporal pole showed the least consistency between the samples. Approximately 91% of the estimates that fell outside the main sample's confidence intervals pertained to estimates in the younger age range (age ≤ 50). All 7 regions that showed a significant effect of age on Δbrain–Δmemory associations were significant in the APOE ε4 non-carrier sample.

Sensitivity analysis using APOE ε4 older subsample.

We conducted a sensitivity analysis to verify whether APOE ε4 carriers show steeper rates of brain and memory decline in old adulthood, without evidence of stronger Δbrain – Δmemory. This analysis was restricted to individuals aged 60 years or more at baseline with APOE ε4 status (n = 2048). APOE ε4 status was associated with steeper memory decline (β = -0.117, $t(2069)$ = -2.9, p = 0.004) and with regional brain decline in 37 regions ($p_{FDR} < 0.05$). The strongest associations were observed in the left and right hippocampus (β_l= -0.209, $t(df)$ = -4.87(2071), p_{FDR} = .0001; β_r = -0.216, $t(df)$ = -4.94(2075), p_{FDR} = .0001), followed by ventricular regions, the left transverse occipital gyrus, and the right amygdala (Supplementary Figure 5). No effects of the APOE ε4 status × age interaction were found on memory decline (β_w = -0.01, edf = 1, p = .50). No APOE ε4 status × age interactions on brain decline were significant (p_{FDR} = 1), including the left and right hippocampus at the uncorrected level ($p_{unc} > .3$). APOE ε4 did not significantly moderate the association between brain decline and memory decline, nor its interaction with age ($p_{FDR} > .2$). This lack of moderation persisted for the hippocampi at the uncorrected level ($p_{unc} > .2$). See complete statistics in the Supporting App. These sensitivity findings are consistent with the main APOE ε4 status results.

Mechanisms behind change – change associations. A post-hoc simulation analysis.

We performed a simulation to explore potential explanations for our empirical findings, particularly the non-linear change – change associations, the moderating effect of age, and the lack of APOE ε4 effects. Using the *sn* (v.2.1.1) package[50], we developed a simplified schematic model where the *observed* brain data resulted from two underlying sources. The first source represented brain aging (true or latent brain change), characterized by a negatively skewed distribution with mean decline indicative of long-term, degenerative changes. The second source was modeled as a Gaussian distribution centered around zero, representing measurement error and other short-term influences[45,51,52]. Memory decline was modeled as linearly related to the brain aging component, plus random Gaussian noise. To explore the moderating effects (or lack thereof) of age and APOE ε4, we adjusted the parameters of the brain aging component, including mean, dispersion (e.g., variability), and skewness.

We generated independent brain aging and measurement error distributions (N = 3700) with the *rsn* function (*sn r-package*). This function is parametrized by location (ξ), scale (ω), and shape (α), which correspond to the distribution's central tendency, dispersion, and skewness, respectively. Note that ξ and ω equate to *mean* (μ) and *SD* (σ) only when α = 0. The measurement error distribution was parametrized with ξ = 0, ω = .8, and α = 0; i.e., a Gaussian distribution with μ = 0 and σ = .8. The main brain aging was defined with ξ = -.8, ω = 1.2, and α = -5; producing a distribution characterized by a mean decline, a standard deviation comparable to that of the measurement error, and a negative (left) skew.

Most individuals exhibited values < 0, with a few showing accelerated decline (more negative values). Memory decline was modeled as linearly related to the brain aging component (r = .3), plus random Gaussian noise (ξ = 0, ω = .8). To test the effect of the mean, and dispersion on the change – change associations, we created distributions with ξ = -.2, -.8, and -1.2 and ω = .6, 1.2, 1.8, respectively. To test the effect of skewness, while maintaining variance (σ^2) constant, we used α = -2, -5, -20 and adjusted ω accordingly to maintain consistent dispersion. We ran 1000 iterations for each model, using GAMs to fit Δbrain – Δmemory associations. The mean trajectories and their 95% *CIs* are presented in Figure 7.

The parameters selected for our simulations are somewhat arbitrary, as they can vary significantly across different regions and populations, e.g., younger vs. older individuals[45]. However, they are roughly comparable to those typically observed in studies of regional brain changes in cognitively healthy older adults. Focusing on hippocampal atrophy, previous research has reported annual hippocampal volume loss rates of -0.84%[52] and -1.05%[45], aligning with the ξ = 0.8% used in our simulations. Assessing variability is more complex, as the results depend on the relationship between variability in latent brain change (brain aging) and measurement noise, which here corresponds to uncertainty in the estimated slopes of brain change. This ratio is influenced by study duration and the number of observations[53]. In prior work, we reported a standard deviation (σ) of 0.6 for yearly hippocampal atrophy rates - compared to $\sigma \approx 0.74$ used in our simulations - and a σ of 1.23 for cross-sectional measurement error. The ratio used in the simulations would roughly match a study design involving three observations over approximately 3 to 3.5 years. Information on skewness is scarce, though we reported mild negative skewness in the distribution of observed brain change[45]. Assuming Gaussian noise, the latent brain change distribution would exhibit more pronounced negative skewness. The degree of skewness attenuation from latent to observed brain change also depends on the variability of the noise. In our simulated data, the brain aging (latent) skewness is γ_1 = -0.85, while the observed skewness is γ_1 = -0.28, which is entirely consistent with our previous estimations. Further research is needed to comprehensively characterize the distributions of brain changes.

Figures and Tables

Supplementary Figure 1. Age distribution of the different datasets.

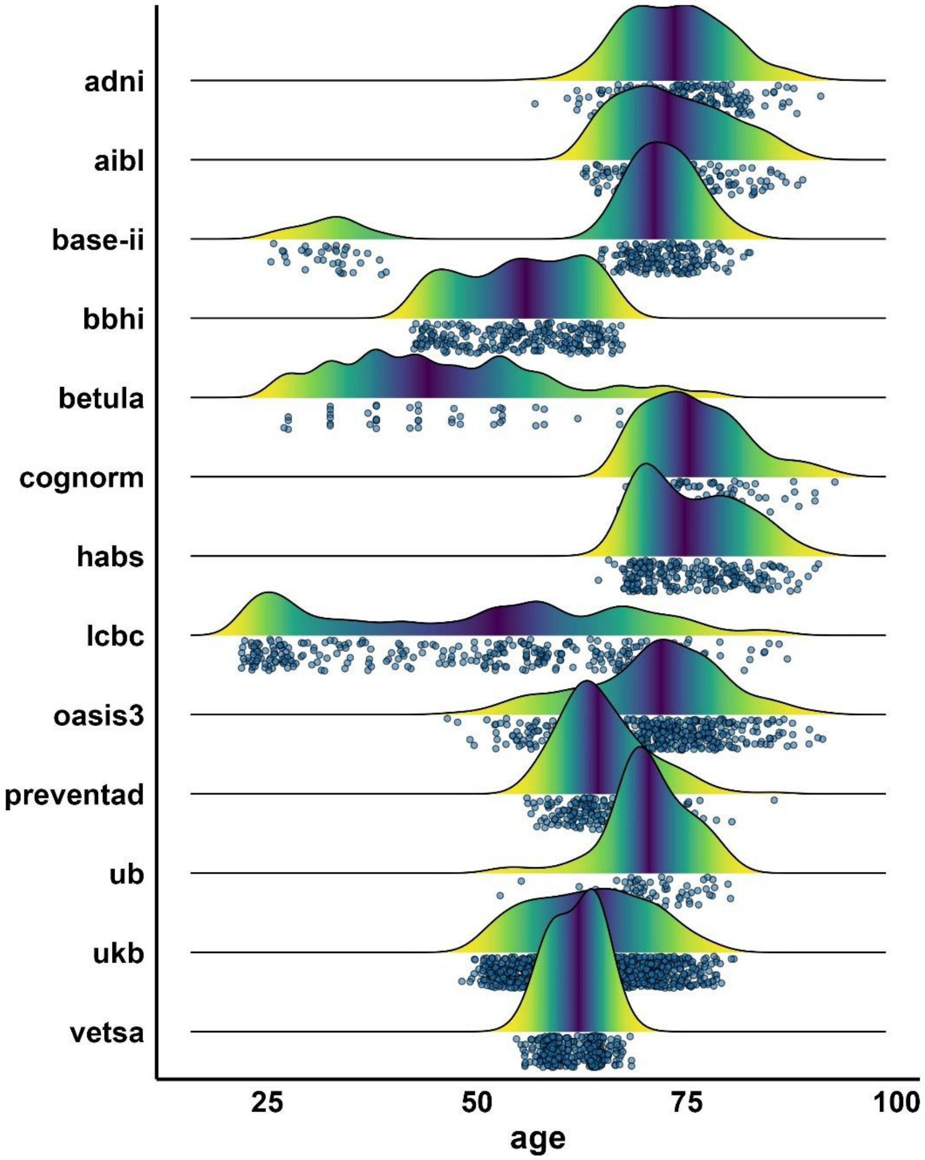

Supplementary Figure 1. Age distributions. Age distributions for the different datasets. Each point represents one individual at its mean age.

Supplementary Figure 2. Hemispheric asymmetry in Memory change – brain change associations

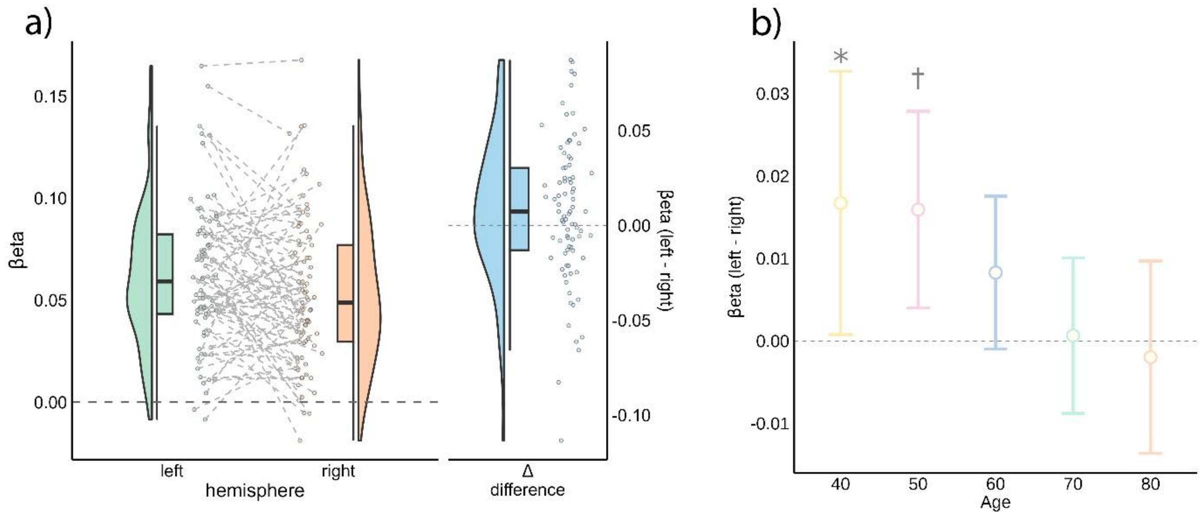

Supplementary Figure 2. Brain change – memory change associations. Hemispheric asymmetry. a) Left panel: Estimates (β_w) for Δbrain - Δmemory associations in the left and right hemispheres per regions. Right panel: hemispheric differences in estimates between left and right hemispheres (paired t-test; $\Delta\beta_w[left\text{-}right] = 0.007$, $t(82) = 1.78$, $p = 0.079$). Estimates were estimated as the density-weighted mean correlation between Δbrain and Δmemory, using the derivative function. Each point represents a region (n = 166). The box represents the interquartile range (*IQR*), line the median, and whiskers extend to 1.5 × *IQR*. **b)** Mean hemispheric difference and 95% *CIs* in regional estimates of Δbrain and Δmemory associations as a function of age. $\Delta\beta_{w[left\text{-}right]}[CI]$ = 0.017[.001, .033], 0.016[.004, .028], 0.008[-.001, .018], 0.001[-.009, .101], -0.002[-.002, 0.010] at 40, 50, 60, 70, and 80 years of age. * denotes uncorrected $p < .05$; † denotes corrected $p_{FDR} < .05$. *pFDR* = False Discovered Rate-corrected p-values.

Supplementary Figure 3. Consensus Clustering results

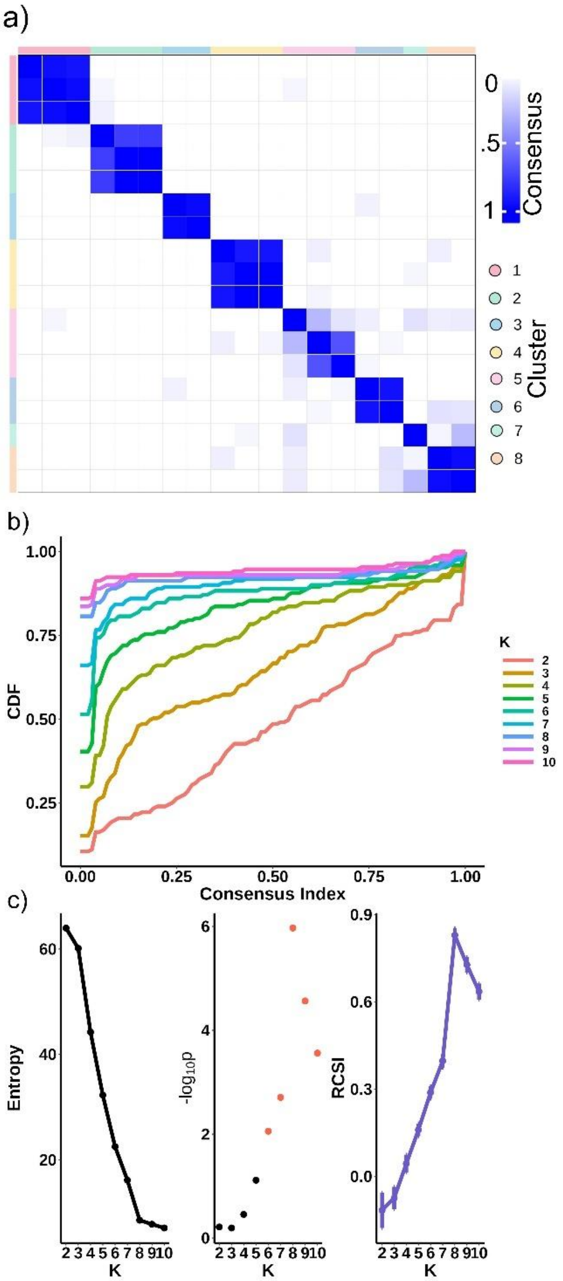

Supplementary Figure 3. Performance metrics of consensus clustering. a) Consensus clustering matrix at the selected solution (*K* = 8). The white-blue scale represents the proportion of iterations two regions have been grouped together in the same cluster. Ideally, values for between-cluster regions are 0, and for within-cluster regions are 1. b) Cumulative density function (*CDF*) plot for consensus clustering visualizing the distribution of consensus values across different *K* values. c) Relative change in the area under the curve (*RCSI*) quantifies how much clustering stability improves as *K* increases. *K* = Number of clusters.

Supplementary Figure 4. APOE ε4 associations with brain change as a function of age.

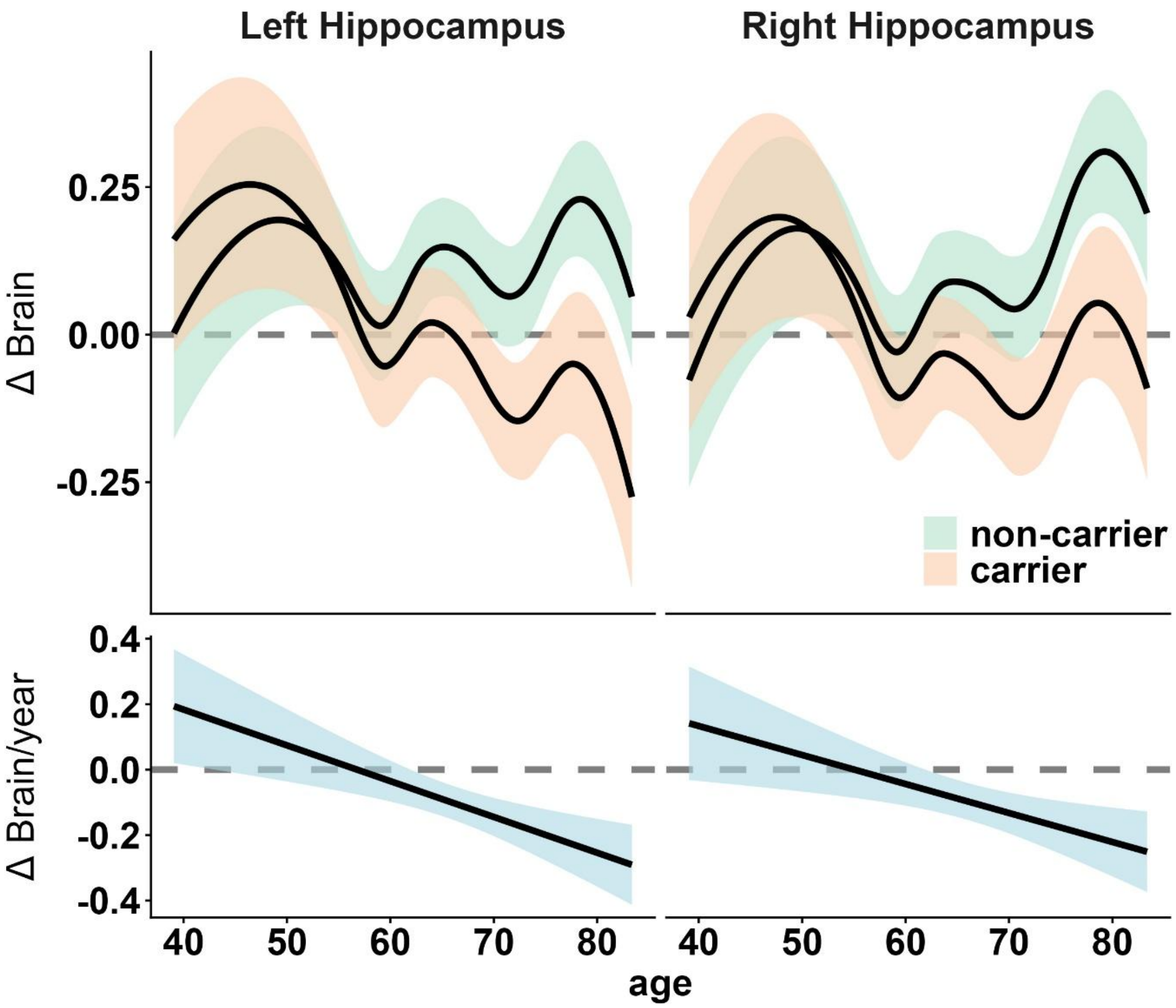

Supplementary Figure 4. APOE ε4 associations with brain change as a function of age. Upper panel: association between APOE ε4 (carriers vs. non-carriers) and brain change as a function of age in the left and right hippocampus (β_{wl} = -.011, *edf* = 1, p_{unc} = 0.01; β_{wr} = -.009, *edf* = 1, p_{unc} = 0.04). Note that age and sex trends are removed based on normative data; thus, brain change should be approximately 0 across age. Lower panel: estimate and 95% *CIs* for the smooth term of APOE ε4 (i.e., additive effect to the reference smooth trajectories of age in APOE ε4 non-carriers. p_{unc} = uncorrected p-value. β_w = Density-weighted betas, using the derivative function.

Supplementary Figure 5. APOE ε4 status effects on regional brain decline using an older subsample.

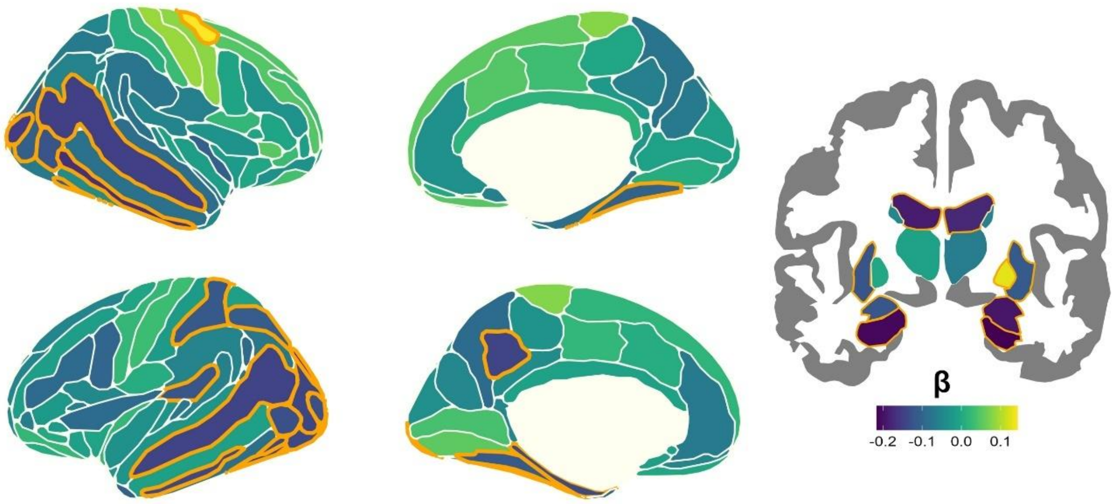

Supplementary Figure 5. APOE ε4 associations with brain change – older subsample. Association between APOE ε4 (carriers, non-carriers) and brain change in a subsample of individuals aged ≥60 years with available APOE ε4 status. Estimates (*β*) for the effect of APOE ε4 status on Δbrain with orange color line representing $p_{FDR} < 0.05$. Δbrain represents atrophy in subcortical and thinning in cortical regions. See Supporting app for complete stats. *pFDR* = False Discovered Rate-corrected p-values.

Supplementary Figure 6. Individual change estimates across harmonization methods.

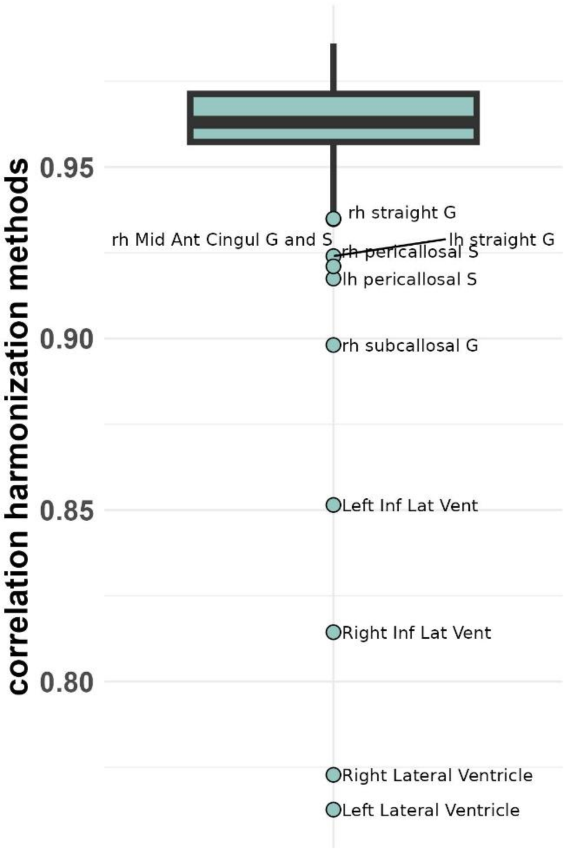

Supplementary Figure 6. Individual change estimates across harmonization methods. Correlation across regions between individual change estimates derived from normative modeling and those obtained using a GAMM-based approach, as in Fjell et al.[46]. The GAMM harmonization model includes sex as fixed factor, age as a smoothing term, and individual, scanner, and dataset as random intercepts. Individual change was estimated as described in the Methods, using the residuals as input data. The boxplot displays the distribution of correlations across regions: the box represents the interquartile range (*IQR*), line the median, and whiskers extend to 1.5 × *IQR*. Outliers are shown as points. Both the mean and median correlation across regions are $r = .96$. Only 5 regions show correlation $r \leq 0.90$, four of which are ventricular features. The lower agreement for ventricular regions is likely due to pronounced age-related increases in variability in ventricular volume, which is modelled in normative models but not in the GAMM-based approach.

Supplementary Table 1. Significant association between brain change and memory change.

Region	FS Nomenclature	β_w	-log10(p)	-log10(p_{fdr})	Partial-R^2
Right Hippocampus	Right-Hippocampus	0.168	3.699	3.699	1.6%
Left Hippocampus	Left-Hippocampus	0.165	3.699	3.699	1.9%
Left Amygdala	Left-Amygdala	0.155	3.699	3.699	1.1%
Right Putamen	Right-Putamen	0.136	2.796	1.541	0.8%
Right Long Insular gyrus	rh_G_Ins_lg_and_S_cent_ins	0.135	3.000	1.683	0.8%
Left Thalamus	Left-Thalamus-Proper	0.135	2.468	1.478	0.8%
Left Inferior Lateral Ventricle	Left-Inf-Lat-Vent	0.132	3.699	3.699	1.1%
Right Amygdala	Right-Amygdala	0.132	3.097	1.722	0.8%
Left Parahippocampal gyrus	lh_G_oc-temp_med-Parahip	0.131	3.097	1.722	0.8%
Left Pericallosal sulcus	lh_S_pericallosal	0.127	3.699	3.699	1.2%
Right Subcallosal gyrus	rh_G_subcallosal	0.114	2.585	1.541	0.9%
Right Anterior Transverse Collateral sulcus	rh_S_collat_transv_ant	0.112	2.585	1.541	0.6%
Right Inferior Temporal gyrus	rh_G_temporal_inf	0.097	2.620	1.541	0.6%
Right Supramarginal gyrus	rh_G_pariet_inf-Supramar	0.095	2.267	1.326	0.6%
Right Straight gyrus	rh_G_rectus	0.093	2.699	1.541	0.6%
Left Inferior temporal gyrus	lh_G_temporal_inf	0.091	2.585	1.541	0.5%
Right Transverse temporal sulcus	rh_S_temporal_transverse	0.081	2.468	1.478	0.6%
Left Caudate	Left-Caudate	0.074	2.745	1.541	0.6%
Right Planum Polare	rh_G_temp_sup-Plan_polar	0.049	2.268	1.326	0.5%

β_w = Density-weighted betas. They were estimated as the density-weighted mean correlation between Δbrain and Δmemory in brain decliners, using the derivative function. p_{FDR} = FDR-corrected p-values. P-values of the smooth variable were obtained using wild bootstrapping, as out-of-the-box GAMM models are anticonservative[54]. See also Figure 1 and Supporting app for complete stats. FS = FreeSurfer. *Partial-R^2* = Difference between (adjusted) variance explained by the full model and a null model without the regressors of interest.

Supplementary Table 2. Significant moderator effect of age on the association between brain change and memory change.

Region	FS Nomenclature	-log10(p)	-log10(p_{fdr})	Partial-R^2
Right Inferior Lateral Ventricle	Right-Inf-Lat-Vent	3.699	3.699	2.0%/1.5%
Left Hippocampus	Left-Hippocampus	3.389	1.771	2.8%/1.0%
Left Short Insular gyrus	lh_G_insular_short	3.397	1.771	1.0%/0.7%
Right Hippocampus	Right-Hippocampus	3.391	1.771	2.6%/1.0%
Right Caudate	Right-Caudate	3.096	1.575	0.6%/0.6%
Right Putamen	Right-Putamen	2.990	1.548	1.5%/0.7%
Left Lateral Ventricle	Left-Lateral-Ventricle	2.691	1.316	1.3%/0.8%

p_{FDR} = FDR-corrected p-values. P-values were obtained using wild bootstrapping, as out-of-the-box GAMM models are anticonservative[54]. See also Figure 2 and Supporting app. FS = FreeSurfer. *Partial-*R^2 = Difference between (adjusted) variance explained by the full model and a null model without the regressors of interest: left estimate includes the brain change and the brain change × age regressors, while the right estimate includes only the interaction R^2.

Supplementary Table 3. Cluster assignment and regional PCA loadings.

Id	Region	FS Nomenclature	PCA1	PCA2	PCA33	PCA4Cl.
1	Left Hippocampus	Left-Hippocampus	0.334	0.101	-0.434	0.0261
2	Right Hippocampus	Right-Hippocampus	0.324	0.111	-0.406	0.0331
3	Right Inferior Temporal gyrus	rh_G_temporal_inf	0.284	-0.374	0.067	0.2744
4	Leftt Inferior Temporal gyrus	lh_G_temporal_inf	0.272	-0.306	0.125	0.1524
5	Left Inferior Lateral Ventricle	Left-Inf-Lat-Vent	0.260	-0.066	-0.487	-0.1251
6	Left Planum Polare	rh_G_temp_sup-Plan_polar	0.255	-0.050	0.197	-0.2496
7	Left Parahippocampal gyrus	lh_G_oc-temp_med-Parahip	0.249	-0.133	0.210	-0.1658
8	Left Thalamus Proper	Left-Thalamus-Proper	0.248	0.304	-0.044	0.2202
9	Right Amygdala	Right-Amygdala	0.232	0.214	0.061	0.2063
10	Right Anterior Transverse Collateral sulcus	rh_S_collat_transv_ant	0.219	-0.150	0.279	-0.2028
11	Right Supramarginal gyrus	rh_G_pariet_inf-Supramar	0.217	-0.339	0.083	0.3304
12	Right Long Insular gyrus	rh_G_Ins_lg_and_S_cent_ins	0.205	-0.014	0.008	-0.2466
13	Right Straight gyrus	rh_G_rectus	0.194	-0.082	0.207	-0.0675
14	Left Amygdala	Left-Amygdala	0.188	0.104	0.087	0.1293
15	Left Caudate	Left-Caudate	0.188	0.427	0.234	0.1222
16	Right Putamen	Right-Putamen	0.163	0.470	0.314	0.1012
17	Right Subcallosal gyrus	rh_G_subcallosal	0.141	0.039	-0.002	-0.3255
18	Right Transverse temporal sulcus	rh_S_temporal_transverse	0.120	-0.095	0.111	-0.2327
19	Left Pericallosal sulcus	lh_S_pericallosal	0.109	0.114	-0.009	-0.5375

Loadings from the first 4 components and cluster assignment for those with significant ($p_{FDR} < .05$) Δbrain – Δmemory associations. See also Figure 3b, c. FS = FreeSurfer. Cl. = Cluster.

Supplementary Table 4. Regional contributions to memory decline

cluster	Basic β(p)	Hippocampus-controlled β(p)	Global decline-controlled β(p)	All clusters in one model β(p)
#1	0.19(<.0001)	--	0.08(.13)	0.09(.00141)
#2	0.11(.0007)	0.03(.354)	-0.01(.790)	0.01(.59905)
#3	0.20(<.0001)	0.11(0.01)	0.09(.0494)	0.08(.2883)
#4	0.13(.001)	0.04(.392)	-0.07(.023)	-0.01(.74433)
#5	0.23(<.0001)	0.16(.002)	0.11(.015)	0.11(0.00844)
#6	0.09(<.0001)	0.06(.012)	0.03(.489)	0.04(.10433)
#7	0.076(.001)	0.05(0.024)	0.03(.012)	0.03(.03584)
#8	0.16(<.0001)	0.10(0.0301)	0.04(.20)	0.07(.01679)

Regional contributions with memory decline. Δbrain – Δmemory associations for the different clusters (K = 8). The different analyses were conducted with weighted GAMMs with dataset as random effect. “Basic” includes only the Δbrain cluster term of interest. In the “hippocampus and global-decline-controlled” models, an extra regressor was added, either the hippocampal cluster (#1) or the global decline as estimated from the PCA. In “all clusters in one” model, we included the eight Δbrain clusters in a single GAMM. All p-values for the smooth variable, except for the last analysis were obtained using bootstrapping (n = 5000 permutations). The p-values the last analysis were obtained as implemented in *mgcv R-package*[49], as bootstrapping was extremely conservative, possibly due to cross-correlation between clusters. See also Figure 3b, c. β_w = Density-weighted betas.

Supplementary Table 5. Initial MRI sample

Dataset	Subjects	Age		Obs. MRI			Time (years)	
	N (m)	M (SD)	range	N	M (SD)	Range	M (SD)	Range
adni	356 (149)	74.0 (6.9)	57.0 -95.2	1206	3.4 (2.2)	1 - 12	2.6 (2.4)	0 – 9.6
aibl	485 (209)	73.1 (6.1)	60.0 -92.0	871	1.8 (1.3)	1 - 5	1.3 (2.1)	0 – 8.0
base-ii	430 (266)	62.0 (17.2)	24.8 – 82.0	753	1.8 (0.5)	1 - 3	1.3 (1.0)	0 – 3.1
bbhi	930 (477)	53.5 (7.1)	40.8 – 67.3	1201	1.3 (0.5)	1 - 2	0.7 (1.1)	0 – 3.0
Betula	303 (150)	62.7 (13.6)	25.0 – 85.0	587	1.9 (0.8)	1 - 3	3.9 (3.3)	0 – 9.0
cognorm	113 (53)	76.3 (6.2)	65.7 – 92.7	423	3.7 (2.0)	1 - 8	5.0 (3.2)	0- 9.5
habs	281 (115)	75.5 (6.1)	64.7 – 90.8	673	2.4 (0.9)	1 - 4	3.5 (2.1)	0 – 8.5
Lcbc	1179 (402)	43.6 (19.7)	20.0 – 93.4	2148	1.8 (1.2)	1 - 7	2.0 (3.1)	0 – 11.5
oasis3	974 (417)	70.5 (8.7)	42.7 – 97.1	2120	2.2 (1.5)	1 - 8	3.0 (3.9)	0 – 15.8
preventad	305 (91)	64.1 (5.0)	55.2 – 85.0	1360	4.5 (1.8)	1 - 7	2.2 (1.5)	0 – 4.7
ub	288 (103)	68.9 (7.0)	38.3 - 89.6	421	1.5 (0.8)	1 - 3	1.0 (1.7)	0 - 5.0
ukb	1287 (627)	63.8 (7.1)	48.2 – 80.6	2574	2.0 (0)	2 - 2	2.3 (0.1)	2 – 2.7
vetsa	731 (731)	61.7 (4.5)	51.1- 71.7	1453	2.0 (0.8)	1 - 3	6.1 (4.9)	0 – 13.4
all	7662 (3790)	61.8 (14.7)	20.0 – 97.1	15790	2.1 (1.3)	1 - 12	2.5 (3.1)	0 – 15.8

Initial MRI sample sociodemographic and observational details. This sample includes both cross-sectional and longitudinal data and was used for MRI data harmonization, i.e., normative modelling *N* = Total number of individuals or observations. *m* = number of males in the sample. NC = Non-carriers. C = Carriers. *M* = mean. *SD* = Standard Deviation. *Obs.* = Observations.

Supplementary Table 6. Initial memory sample

Dataset	Subjects	Age		Obs. Memory			Time (years)	
	N (m)	M (SD)	Range	N	M (SD)	Range	M (SD)	Range
adni	904 (405)	74.0 (6.7)	55.1 – 92.3	3824	4.2 (2.9)	1 - 15	3.4 (3.2)	0 – 15.0
aibl	615 (270)	73.6 (6.2)	60.0 – 94.0	1239	2.0 (1.3)	1 - 5	1.6 (2.2)	0 – 7.0
base-ii	1899 (936)	64.0 (16.9)	23.6 – 91.2	3133	1.6 (0.7)	1 - 3	2.7 (2.6)	0 – 6.5
bbhi	973 (500)	53.5 (7.2)	29.5 – 67.3	1276	1.3 (0.5)	1 - 2	0.7 (1.1)	0 – 3.0
betula	337 (168)	57.1 (11.4)	25.0 – 87.9	1563	4.6 (1.9)	1 - 7	17.6 (8.9)	0 – 29.0
cognorm	114 (54)	76.1 (6.2)	67.0 – 92.6	667	5.9 (1.8)	1 - 7	5.2 (1.9)	0- 6.9
habs	287 (118)	75.5 (6.1)	64.5 – 90.7	1251	4.4 (2.0)	1 - 6	3.5 (2.1)	0 – 8.5
Lcbc	939 (303)	39.7 (17.5)	20.0 – 93.4	1442	1.5 (0.8)	1 - 4	2.1 (3.2)	0 – 11.5
oasis3	1067 (463)	71.4 (8.6)	45.7 – 96.2	6561	6.1 (4.6)	1 - 30	7.0 (5.5)	0 – 31.6
preventad	306 (91)	64.4 (5.1)	55.2 – 85.9	1057	3.5 (1.8)	1 - 6	2.1 (1.5)	0 – 4.5
ub	161 (57)	69.3 (5.2)	51.3 – 85.8	298	1.9 (0.9)	1 - 3	1.8 (2.0)	0 - 5.2
ukb	33890 (16456)	65.3 (7.6)	47.3 – 82.8	36520	1.1 (0.3)	1 - 2	0.2 (0.6)	0 – 3.0
vetsa	1592 (1592)	61.5 (3.9)	52.1 – 71.1	3617	2.3 (0.8)	1 - 3	7.6 (4.8)	0 – 14.4
all	43084 (21413)	64.8 (9.7)	20.0 – 96.2	62448	1.4 (1.4)	1 - 30	1.1 (3.0)	0 – 31.6

Initial memory sample sociodemographic and observational details. This sample includes both cross-sectional and longitudinal data and was used for episodic memory data harmonization, i.e., normative modelling *N* = Total number of individuals or observations. m = number of males in the sample. NC = Non-carriers. C = Carriers. *M* = mean. *SD* = Standard Deviation. *Obs.* = Observations.

Supplementary Table 7. Data availability

Sample	Link	PI and/or Admin Contact	IRB
		Longitudinal Aging Dataset	
ADNI	https://adni.loni.usc.edu/ (O)	Weiner MW; michael.weiner@ucsf.edu (PI) ida@loni.usc.edu (AC)	Approved by the Institutional Review Boards of all of the participating institutions
AIBL	https://aibl.csiro.au/research/ (O)	Christopher Rowe; christopher.rowe@austin.org.au (PI)	Institutional ethics committees of Austin Health, StVincent's Health, Hollywood
BASE-II	https://www.base2.mpg.de/en (R)	Lindenberger U (lindenberger@mpib-berlin.mpg.de), Düzel E (e.duzel@ucl.ac.uk), Kühn S (kuehn@mpib-berlin.mpg.de) (PI); Ludmila Muller (lmueller@mpib-berlin.mpg.de)	Ethics Committee of the Max-Planck-Institute
BBHI	https://bbhi.cat/en (R)	Alvaro Pascual-Leone (apleone@hsl.harvard.edu)(PI); bbhi@guttmann.com (AC)	Research institutional review board of the Institut Guttmann
BETULA	https://www.umu.se/en/research/projects/betula---aging-memory-and-dementia/ (R)	Lars Nyberg; lars.nyberg@umu.se (PI)	Regional Ethical Vetting Board at Umeå University
COGNORM	https://www.med.uio.no/klinmed/english/research/groups/delirium/index.html; http://www.oslobrains.no (R)	Leiv Otto Watne (l.o.watne@medisin.uio.no) (PI); Anders Martin Fjell; a.m.fjell@psykologi.uio.no (PI)	Norwegian Regional Committees for Medical and Health Research Ethics and the Data Protector Officer at Oslo University Hospital
HABS	https://habs.mgh.harvard.edu (O)	Reisa Sperling; reisa@rics.bwh.harvard.edu (PI); habs@mgh.harvard.edu (AC)	Partners Healthcare Human Research Committee
LCBC	http://www.oslobrains.no (R)	Kristine B. Walhovd; k.b.walhovd@psykologi.uio.no (PI)	Norwegian Regional Committee for Medical and Health Research Ethic; Regional Ethical Committee of South Norway
OASIS3	https://www.oasis-brains.org/ (O)	Pamela J. LaMontagne; pjlamontagne@wustl.edu (PI); Daniel Marcus; dmarcus@wustl.edu (PI); https://www.oasis-brains.org/#contact (AC)	Institutional Review Board of Washington University School of Medicine

preventAD	https://prevent-alzheimer.net; https://openpreventad.loris.ca/ (O)	Jennifer Tremblay; jennifer.tremblay-mercier@douglas.mcgill.ca (PI); https://openpreventad.loris.ca/contact/ (AC)	The McGill Institutional Review Board and the Douglas Mental Health University Institute Research Ethics Board
UB	http://www.ub.edu/bbslab/bbslab/ (R)	David Bartrés-Faz; dbartres@ub.edu (PI)	Comisión de Bioética de la Universidad de Barcelona and Hospital Clinic
UKB	https://www.ukbiobank.ac.uk/ (O)	Rory Collins (rory.collins@ndph.ox.ac.uk) (PI); access@ukbiobank.ac.uk (AC)	Northwest Multi-Center Research Ethics Committee [MREC]; https://www.ukbiobank.ac.uk/the-ethics-and-governance-council
VETSA	https://www.vetsatwins.org/ (O	William S. Kremen (wkremen@ucsd.edu)(PI); https://www.vetsatwins.org/for-researchers/ (AC)	VETSA and VET Registry Data Security Policies, UCSD Human Subject Committee

Data availability, contact and principal investigator information, and ethical approval for the different datasets used. PI = Principal Investigator. AC = Administrative contact. IRB = Institutional Review Boards. O = Openly available. Automatic or semi-automatic data agreements. Fees may apply (e.g. UKB). R = Restricted. Ad-hoc permission is required. Contact PI or AC for specific details on securing access to data.

Supplementary Table 8. Memory measures.

Dataset	*Subject (Obs.)*	Memory Tests
ADNI	904 (3824)	ADNI-MEM[1]
AIBL	615 (1239)	Logical memory short delay recall
		Logical memory long delay recall
BASE-II	1899 (3133)	VLMT short delay recall
		VLMT long delay recall
		VLMT learning (sum across trials)
		Scene encoding task (hit – fa)[2]
		Face-profession task (hits – new fa) [2]
		Face-profession task (hits – rear fa) [2]
		Object location task (short delay recall) [2]
BBHI	966 (1266)	RAVLT learning (sum across trials)
		RAVLT long delay recall
		RAVLT short delay recall
BETULA	337 (1563)	Recall of sentences[3]
COGNORM	114 (667)	CERAD short delay recall
		CERAD long delay recall
HABS	615 (1239)	Logical memory short delay recall
		Logical memory long delay recall
		SRT delayed recall
		SRT total recall
		FCsrt free recall
LCBC	938 (1442)	CVLT short delay recall
		CVLT long delay recall
		CVLT learning (sum across trials)
OASIS3	1067 (6561)	Logical memory immediate
		Logical memory delayed
		SRT delayed recall
		SRT total recall
		WMS associate learning summary score
preventAD	306 (1057)	RBANS list recall
		RBANS list learning (sum across trials)
		RBANS story immediate memory
		RBANS story delayed recall
		RBANS figure recall total score
UB	161 (298)	RAVLT learning (sum across trials)
		RAVLT long delay recall
UKB	33,890 (36,520)	PAL[4]
VETSA	1592 (3617)	CVLT short delay recall
		CVLT long delay recall
		CVLT learning (sum across trials)

Memory measures selected in each dataset. A PC was estimated on the first time point when multiple memory measures were available. Subs (Obs.). Subjects and Observations with memory from the initial MRI sample. MMSE = Mini-mental State Examination. RAVLT = Rey Auditory Verbal Learning Test; CVLT = California Verbal Learning Test; PAL = Paired associate

learning (#20197 UKB field); Logical memory = Memory subtest of the Wechsler Memory Scale. CERAD = Consortium to Establish a Registry for Alzheimer's Disease (CERAD) Word List Memory test. ADAS = Alzheimer Disease Assessment Scale. VMLT = Verbal Learning and Memory test. SRT = Buschke Selective Reminding Task. RBANS = Repeatable Battery for Assessment of Neuropsychological Status. Story recall = Story recall and recognition task of episodic memory from Wechsler Neuropsychological Battery. FRsrt = Free and Cued selective reminding test. WMS = Weschler Memory scale. FA = False alarms. [1]ADNI-MEM score was computed developed by [55] and consists of a composite score of memory which includes measures from RAVLT (learning trials, list, recognition and recalls), ADAS (learning trials, recall, and recognitions), MMSE words, and Logical memory. [2]See [5,6] for more details. [3]See [21] for more details. [4]See https://biobank.ndph.ox.ac.uk/showcase/refer.cgi?id=2561 for more information on PAL.

Supplementary Table 9. Scanner acquisition parameters.

Dataset	Scanner	Field	Sequence parameters
ADNI	Multisite (n > 50)	1.5/ 3.0	See https://adni.loni.usc.edu/methods/documents/mri-protocols/
AIBL	Avanto Siemens	1.5	MPRAGE. TR: 2300 ms; TE: 2.98 ms, TI: 900 ms; flip angle 9°, slice thickness: 1.25 mm, FoV: 240 x 256, 160 slices.
	Verio Siemens	3.0	MPRAGE. TR: 2300 ms; TE: 2.98 ms, TI: 900 ms; flip angle 9°, slice thickness: 1.25 mm, FoV: 240 x 256, 160 slices.
	Tim Trio Siemens	3.0	MPRAGE. TR: 2300 ms; TE: 2.98 ms, TI: 900 ms; flip angle 9°, slice thickness: 1.25 mm, FoV 240 x 256, 160 slices.
BASE-II	Tim Trio Siemens	3.0	MPRAGE. TR: 2500 ms; TE: 4.77 ms, TI: 1100 ms; flip angle 7°, slice thickness: 1.0 mm, FoV 256 x 256, 176 slices.
BBHI	MAGNETOM Prisma Siemens	3.0	MPRAGE. TR: 2400 ms; TE: 2.22 ms, TI: 1000 ms; flip angle 8°, slice thickness: 1.0 mm, FoV 250 x 250, 208 slices.
BETULA	Discovery GE	3.0	3D FSPGR. TR: 8.19 ms; TE: 3.2ms, TI: 450 ms; flip angle 12°, slice thickness: 1 mm, FoV: 250 x 250, 180 slices.
COGNORM	Siemens Avanto		MPRAGE. TR: 2400 ms; TE: 3.79 ms, TI: 1000 ms; flip angle 8°, slice thickness: 1.2 mm, FoV 240 x 240, 160 slices.
	Siemens Prisma	3.0	MPRAGE. TR: 2400 ms; TE: 2.22 ms, TI: 1000 ms; flip angle 8°, slice thickness: 0.8 mm, FoV 240 x 256, 208 slices, iPat = 2.
HABS[c]	Tim Trio Siemens	3.0	MPRAGE. TR: 2300 ms; TE: 2.98 ms, TI: 900 ms; flip angle 9°, slice thickness: 1.2 mm, FoV 240 x 256, 160 slices.
			MPRAGE. TR: 2200 ms; TE: 1.5/3.4/5.2/7.0 ms, TI: 1100 ms; flip angle 7°, slice thickness: 1.2 mm, FoV: 228 x 228, 144 slices, Multi-echo = x4.
LCBC	Avanto Siemens	1.5	MPRAGE. TR: 2400 ms; TE: 3.79 ms, TI: 1000 ms; flip angle 8°, slice thickness: 1.2 mm, FoV 240 x 240, 160 slices.
	Siemens Skyra	3.0	MPRAGE. TR: 2300 ms; TE: 2.98 ms, TI: 850 ms; flip angle 8°, slice thickness: 1 mm, FoV: 256 x 256, 176 slices.
	Siemens Prisma	3.0	MPRAGE. TR: 2400 ms; TE: 2.22 ms, TI: 1000 ms; flip angle 8°, slice thickness: 0.8 mm, FoV 240 x 256, 208 slices, iPat = 2.
OASIS3	Siemens Vision	1.5	MPRAGE. TR: 9,7 ms; TE: 4.0 ms, TI: 20 ms; flip angle 10°, slice thickness: 1.25 mm, FoV: 256 x 256, 160 slices.
	Siemens Sonata	1.5	MPRAGE. TR: 9,7 ms; TE: 3.9 ms, TI: 20 ms; flip angle 15°, slice thickness: 1 mm, FoV: 224 x 256, 160 slices.
	Tim Trio Siemens	3.0	MPRAGE. TR: 2400 ms; TE: 3.1 ms, TI: 1000 ms; flip angle 8°, slice thickness: 1 mm, FoV 256 x 256, 176 slices.
	Magnetom Vida Siemens	3.0	MPRAGE. TR: 2300 ms; TE: 2.3 ms, TI: 900 ms; flip angle 9°, slice thickness: 1.2 mm, FoV 240 x 256, 176 slices.
	Siemens BioGraph mMR	3.0	MPRAGE. TR: 2300 ms; TE: 2.3 ms, TI: 900 ms; flip angle 9°, slice thickness: 1.2 mm, FoV 240 x 256, 176 slices.
preventAD	Tim Trio Siemens	3.0	MPRAGE. TR: 2300 ms; TE: 2.98 ms, TI: 900 ms; flip angle 9°, slice thickness: 1 mm, FoV 240 x 256, 176 slices.
UB	Tim trio Siemens	3.0	MPRAGE. TR: 2400 ms; TE: 2.98 ms, TI: 900 ms; flip angle 9°, slice thickness: 1 mm, FoV: 256 x 256, 240 slices.
UKB	Siemens Skyra[a]	3.0	MPRAGE. TR: 2000 ms; TE: - ms, TI: 880 ms; flip angle -, slice thickness: 1 mm, FoV: 208 x 256, 256 slices.

	Siemens Avanto	1.5	MPRAGE. TR: 1000 ms; TE: 3.31 ms, TI: 1000 ms; flip angle 7°, slice thickness: 1.33 mm, FoV 256 x 256, 128 slices
	Siemens Symphony	1.5	MPRAGE. TR: 1000 ms; TE: 3.31 ms, TI: 1000 ms; flip angle 7°, slice thickness: 1.33 mm, FoV 256 x 256, 128 slices
VETSA	Discovery 750× GE[d]	3.0	3D FSPGR. TR: 8.084 ms; TE: 3.164ms, TI: 600 ms; flip angle 8°, slice thickness: 1.2 mm, FoV: 256 x 256, 176 slices.
	Tim Trio Siemens	3.0	MPRAGE. TR: 2170 ms; TE: 4.33 ms, TI: 1100 ms; flip angle 7°, slice thickness: 1.2 mm, FoV 256 x 256, 160 slices.

Scanner Parameters for each dataset. TR = Repetition Time; TE = Echo Time; TI = inversion time; FoV = Field of View, iPat = in-plane acceleration. [a,c,d]Two matched scanners. [b]Several matched scanners.

References

1. Walhovd, K. B. *et al.* Neurodevelopmental origins of lifespan changes in brain and cognition. *Proc. Natl. Acad. Sci. U.S.A.* **113**, 9357–9362 (2016).
2. Nyberg, L. *et al.* Longitudinal evidence for diminished frontal cortex function in aging. *Proc. Natl. Acad. Sci. U.S.A.* **107**, 22682–22686 (2010).
3. Rajaram, S. *et al.* The Walnuts and Healthy Aging Study (WAHA): Protocol for a Nutritional Intervention Trial with Walnuts on Brain Aging. *Front Aging Neurosci* **8**, (2017).
4. Vidal-Piñeiro, D. *et al.* Task-dependent activity and connectivity predict episodic memory network-based responses to brain stimulation in healthy aging. *Brain Stimul* **7**, 287–296 (2014).
5. Bertram, L. *et al.* Cohort profile: The Berlin Aging Study II (BASE-II). *Int J Epidemiol* **43**, 703–712 (2014).
6. Gerstorf, D. *et al.* Editorial. *Gerontology* **62**, 311–315 (2016).
7. Walhovd, K. B. *et al.* Healthy minds 0-100 years: Optimising the use of European brain imaging cohorts ('Lifebrain'). *Eur. Psychiatry* **50**, 47–56 (2018).
8. Idland, A.-V. *et al.* Biomarker profiling beyond amyloid and tau: cerebrospinal fluid markers, hippocampal atrophy, and memory change in cognitively unimpaired older adults. *Neurobiol. Aging* **93**, 1–15 (2020).
9. Mueller, S. G. *et al.* The Alzheimer's disease neuroimaging initiative. *Neuroimaging Clin. N. Am.* **15**, 869–877, xi–xii (2005).
10. Ellis, K. A. *et al.* The Australian Imaging, Biomarkers and Lifestyle (AIBL) study of aging: methodology and baseline characteristics of 1112 individuals recruited for a longitudinal study of Alzheimer's disease. *Int Psychogeriatr* **21**, 672–687 (2009).

11. Cattaneo, G. *et al.* The Barcelona Brain Health Initiative: A Cohort Study to Define and Promote Determinants of Brain Health. *Front. Aging Neurosci.* **10**, (2018).
12. Dagley, A. *et al.* Harvard Aging Brain Study: dataset and accessibility. *Neuroimage* **144**, 255–258 (2017).
13. Miller, K. L. *et al.* Multimodal population brain imaging in the UK Biobank prospective epidemiological study. *Nature Neuroscience* **19**, 1523–1536 (2016).
14. Breitner, J. C. S., Poirier, J., Etienne, P. E. & Leoutsakos, J. M. Rationale and Structure for a New Center for Studies on Prevention of Alzheimer's Disease (StoP-AD). *J Prev Alzheimers Dis* **3**, 236–242 (2016).
15. Tremblay-Mercier, J. *et al.* Open science datasets from PREVENT-AD, a longitudinal cohort of pre-symptomatic Alzheimer's disease. *Neuroimage Clin* **31**, 102733 (2021).
16. LaMontagne, P. J. *et al.* OASIS-3: Longitudinal Neuroimaging, Clinical, and Cognitive Dataset for Normal Aging and Alzheimer Disease. 2019.12.13.19014902 Preprint at https://doi.org/10.1101/2019.12.13.19014902 (2019).
17. Kremen, W. S., Franz, C. E. & Lyons, M. J. VETSA: The Vietnam Era Twin Study of Aging. *Twin research and human genetics : the official journal of the International Society for Twin Studies* **16**, 399 (2012).
18. Petersen, R. C. *et al.* Alzheimer's Disease Neuroimaging Initiative (ADNI): clinical characterization. *Neurology* **74**, 201–209 (2010).
19. Cattaneo, G. *et al.* The Barcelona Brain Health Initiative: Cohort description and first follow-up. *PLoS One* **15**, e0228754 (2020).
20. Nilsson, L.-G. *et al.* Betula: A Prospective Cohort Study on Memory, Health and Aging. *Aging, Neuropsychology, and Cognition* **11**, 134–148 (2004).

21. Nilsson, L.-Gör. *et al.* The betula prospective cohort study: Memory, health, and aging. *Aging, Neuropsychology, and Cognition* **4**, 1–32 (1997).
22. Idland, A.-V. *et al.* CSF neurofilament light levels predict hippocampal atrophy in cognitively healthy older adults. *Neurobiology of Aging* **49**, 138–144 (2017).
23. Sajjad, M. U. *et al.* Cerebrospinal Fluid Levels of Interleukin-8 in Delirium, Dementia, and Cognitively Healthy Patients. *J Alzheimers Dis* **73**, 1363–1372 (2020).
24. Dagley, A. *et al.* Harvard Aging Brain Study: Dataset and accessibility. *NeuroImage* **144**, 255–258 (2017).
25. Fjell, A. M. *et al.* Sleep duration and brain atrophy – phenotypic associations and genotypic covariance. *Nature Human Behaviour* (2023).
26. LaMontagne, P. *et al.* OASIS-3: Longitudinal Neuroimaging, Clinical, and Cognitive Dataset for Normal Aging and Alzheimer Disease. *medRxiv* 2019.12.13.19014902 (2019) doi:10.1101/2019.12.13.19014902.
27. Morris, J. C. The Clinical Dementia Rating (CDR): current version and scoring rules. *Neurology* **43**, 2412–2414 (1993).
28. Tremblay-Mercier, J. *et al.* Open science datasets from PREVENT-AD, a longitudinal cohort of pre-symptomatic Alzheimer’s disease. *Neuroimage Clin* **31**, 102733 (2021).
29. Abellaneda-Pérez, K. *et al.* Age-related differences in default-mode network connectivity in response to intermittent theta-burst stimulation and its relationships with maintained cognition and brain integrity in healthy aging. *Neuroimage* **188**, 794–806 (2019).
30. Uribe, C. *et al.* Patterns of cortical thinning in nondemented Parkinson’s disease patients. *Mov Disord* **31**, 699–708 (2016).

31. Guggenheim, J. A., Williams, C., & UK Biobank Eye and Vision Consortium. Role of Educational Exposure in the Association Between Myopia and Birth Order. *JAMA Ophthalmol* **133**, 1408–1414 (2015).

32. Kremen, W. S., Franz, C. E. & Lyons, M. J. Current Status of the Vietnam Era Twin Study of Aging (VETSA). *Twin Res Hum Genet* **22**, 783–787 (2019).

33. Kremen, W. S. *et al.* Genes, environment, and time: the Vietnam Era Twin Study of Aging (VETSA). *Twin Res Hum Genet* **9**, 1009–1022 (2006).

34. Gorgolewski, K. J. *et al.* The brain imaging data structure, a format for organizing and describing outputs of neuroimaging experiments. *Sci Data* **3**, 160044 (2016).

35. Routier, A. *et al.* Clinica: An Open-Source Software Platform for Reproducible Clinical Neuroscience Studies. *Front Neuroinform* **15**, 689675 (2021).

36. Samper-González, J. *et al.* Reproducible evaluation of classification methods in Alzheimer's disease: Framework and application to MRI and PET data. *NeuroImage* **183**, 504–521 (2018).

37. Reuter, M., Schmansky, N. J., Rosas, H. D. & Fischl, B. Within-subject template estimation for unbiased longitudinal image analysis. *Neuroimage* **61**, 1402–1418 (2012).

38. Dale, A. M., Fischl, B. & Sereno, M. I. Cortical surface-based analysis. I. Segmentation and surface reconstruction. *Neuroimage* **9**, 179–194 (1999).

39. Fischl, B., Sereno, M. I. & Dale, A. M. Cortical surface-based analysis. II: Inflation, flattening, and a surface-based coordinate system. *Neuroimage* **9**, 195–207 (1999).

40. Reuter, M., Rosas, H. D. & Fischl, B. Highly accurate inverse consistent registration: A robust approach. *NeuroImage* **53**, 1181–1196 (2010).

41. Rutherford, S. *et al.* The normative modeling framework for computational psychiatry. *Nat Protoc* **17**, 1711–1734 (2022).

42. Rutherford, S. *et al.* Charting brain growth and aging at high spatial precision. *eLife* **11**, e72904 (2022).

43. Van Rossum, G. & Drake, F. L. *Python 3 Reference Manual*. (CreateSpace, Scotts Valley, CA, 2009).

44. Kia, S. M. *et al.* Hierarchical Bayesian Regression for Multi-site Normative Modeling of Neuroimaging Data. in *Medical Image Computing and Computer Assisted Intervention – MICCAI 2020* (eds. Martel, A. L. et al.) 699–709 (Springer International Publishing, Cham, 2020). doi:10.1007/978-3-030-59728-3_68.

45. Vidal-Piñeiro, D. *et al.* Reliability of structural brain change in cognitively healthy adult samples. 2024.06.03.592804 Preprint at https://doi.org/10.1101/2024.06.03.592804 (2024).

46. Fjell, A. M. *et al.* Reevaluating the role of education on cognitive decline and brain aging in longitudinal cohorts across 33 Western countries. *Nat Med* (2025) doi:10.1038/s41591-025-03828-y.

47. Bates, D., Mächler, M., Bolker, B. & Walker, S. Fitting Linear Mixed-Effects Models Using lme4. *Journal of Statistical Software* **67**, 1–48 (2015).

48. Kuznetsova, A., Brockhoff, P. B. & Christensen, R. H. B. lmerTest Package: Tests in Linear Mixed Effects Models. *Journal of Statistical Software* **82**, 1–26 (2017).

49. Wood, S. N. *Generalized Additive Models: An Introduction with R*. (Chapman and Hall/CRC, 2017).

50. Azzalini, A. A. *The R Package sn: The Skew-Normal and Related Distributions Such as the Skew-t and the SUN (Version 2.1.1).* (Università degli Studi di Padova, Italia, 2023).

51. Bråthen, A. C. S. *et al.* Cognitive and hippocampal changes weeks and years after memory training. *Sci Rep* **12**, 7877 (2022).

52. Erickson, K. I. *et al.* Exercise training increases size of hippocampus and improves memory. *Proc. Natl. Acad. Sci. U.S.A.* **108**, 3017–3022 (2011).

53. Rast, P. & Hofer, S. M. Longitudinal design considerations to optimize power to detect variances and covariances among rates of change: simulation results based on actual longitudinal studies. *Psychol Methods* **19**, 133–154 (2014).

54. Simonsohn, U. Interacting With Curves: How to Validly Test and Probe Interactions in the Real (Nonlinear) World. *Advances in Methods and Practices in Psychological Science* **7**, 25152459231207787 (2024).

55. Crane, P. K. *et al.* Development and assessment of a composite score for memory in the Alzheimer's Disease Neuroimaging Initiative (ADNI). *Brain Imaging Behav* **6**, 502–516 (2012).
