# Supplementary material for: Vulnerability to memory decline in aging revealed by a mega-analysis of structural brain change
Source: Nat Commun. 2025 Nov 21;16:11488. doi: 10.1038/s41467-025-66354-y (PMC12749543; doi:10.1038/s41467-025-66354-y)
Supplement: Supplementary file 2 — Reporting Summary [file 41467_2025_66354_MOESM2_ESM.pdf]

nature portfolio

Corresponding author(s): Didac Vidal Pineiro

Last updated by author(s): Oct 24, 2025

# Reporting Summary

Nature Portfolio wishes to improve the reproducibility of the work that we publish. This form provides structure for consistency and transparency in reporting. For further information on Nature Portfolio policies, see our Editorial Policies and the Editorial Policy Checklist.

## Statistics

For all statistical analyses, confirm that the following items are present in the figure legend, table legend, main text, or Methods section.

| n/a | Confirmed | |
|---|---|---|
| ☐ | ☒ | The exact sample size (*n*) for each experimental group/condition, given as a discrete number and unit of measurement |
| ☐ | ☒ | A statement on whether measurements were taken from distinct samples or whether the same sample was measured repeatedly |
| ☐ | ☒ | The statistical test(s) used AND whether they are one- or two-sided<br>*Only common tests should be described solely by name; describe more complex techniques in the Methods section.* |
| ☐ | ☒ | A description of all covariates tested |
| ☐ | ☒ | A description of any assumptions or corrections, such as tests of normality and adjustment for multiple comparisons |
| ☐ | ☒ | A full description of the statistical parameters including central tendency (e.g. means) or other basic estimates (e.g. regression coefficient) AND variation (e.g. standard deviation) or associated estimates of uncertainty (e.g. confidence intervals) |
| ☐ | ☒ | For null hypothesis testing, the test statistic (e.g. *F*, *t*, *r*) with confidence intervals, effect sizes, degrees of freedom and *P* value noted<br>*Give P values as exact values whenever suitable.* |
| ☒ | ☐ | For Bayesian analysis, information on the choice of priors and Markov chain Monte Carlo settings |
| ☒ | ☐ | For hierarchical and complex designs, identification of the appropriate level for tests and full reporting of outcomes |
| ☐ | ☒ | Estimates of effect sizes (e.g. Cohen's *d*, Pearson's *r*), indicating how they were calculated |

*Our web collection on statistics for biologists contains articles on many of the points above.*

## Software and code

Policy information about availability of computer code

| | |
|---|---|
| Data collection | Structural T1-weighted (T1w) MPRAGE and FSPGR scans were collected using 1.5 and 3T MRI scanners. All data is legacy data (not acquired directly by the researchers). Over >50 different scans were used. See Supplementary Table 9 for detail on all the scanners |
| Data analysis | Data preprocessed on the Colossus processing cluster, part of the Tjenester for Sensitive Data (TSD) using bash for parallelization purposes<br>MRI preprocessing: Clinica software (v.0.9.2) for bids conversion; MRI preprocessing with Longitudinal FreeSurfer stream (v.7.1.0).<br>MRI normative modelling: with the PCNtoolkit (0.30.post2), in Python3 environment (version 3.9.5).<br>Remaining analyses were carried in R environment (version 4.2.1).<br>Core packages used: visualisation (ggplot2[v.4.0.0], ggseg [v.1.6.5]); modelling (lme4 [v. 1.1-37], lmerTest [v.3.1-3], mgvc [v. 1.9-3], gratia [v. 0.11-1], m3c [v. 1.30.0] , sn [v. 2.1.1])<br>Code deposited at: https://github.com/daidak/memory-brain-change (https://doi.org/10.5281/zenodo.17433776) |

For manuscripts utilizing custom algorithms or software that are central to the research but not yet described in published literature, software must be made available to editors and reviewers. We strongly encourage code deposition in a community repository (e.g. GitHub). See the Nature Portfolio guidelines for submitting code & software for further information.

# Data

Policy information about availability of data

All manuscripts must include a data availability statement. This statement should provide the following information, where applicable:

- Accession codes, unique identifiers, or web links for publicly available datasets
- A description of any restrictions on data availability
- For clinical datasets or third party data, please ensure that the statement adheres to our policy

The raw data were gathered from 13 different third-party datasets. As such, there are restrictions on data availability. Data will be made available for purposes of peer review, inside UiO's secure server, within the terms of a data use agreement and if compliant with ethical and legal requirements. Group-level estimates generated in this study are provided in the Supporting app (https://vidalpineiro.shinyapps.io/brain_mem_change/).
Different agreements are required for each dataset. Most dataset are openly available with prespecified data usage agreements. For some datasets, such as UKB, fees may apply. Requests for Lifebrain cohorts (LCBC, Umeå, UB) and COGNORM, should be submitted to the corresponding principal investigator. See detailed data availability and contact details for each dataset below.

LCBC: Kristine B. Walhovd; k.b.walhovd@psykologi.uio.no (PI); http://www.oslobrains.no
Betula: Lars Nyberg; lars.nyberg@umu.se (PI); https://www.umu.se/en/research/projects/betula---aging-memory-and-dementia/;
UB: David Bartrés-Faz; dbartres@ub.edu (PI); http://www.ub.edu/bbslab/bbslab/
BASE-II: Lindenberger U (lindenberger@mpib-berlin.mpg.de), Düzel E (e.duzel@ucl.ac.uk), Kühn S ( kuehn@mpib-berlin.mpg.de) (PI); Ludmila Muller (lmueller@mpib-berlin.mpg.de); https://www.base2.mpg.de/en
COGNORM: Leiv Otto Watne (l.o.watne@medisin.uio.no) (PI); Anders Martin Fjell; a.m.fjell@psykologi.uio.no (PI); https://www.med.uio.no/klinmed/english/research/groups/delirium/index.html
Alzheimer's Disease Neuroimaging Initiative (ADNI): Weiner MW; michael.weiner@ucsf.edu (PI); ida@loni.usc.edu (AC); https://adni.loni.usc.edu
AIBL: Christopher Rowe; christopher.rowe@austin.org.au (PI); https://aibl.csiro.au/research/
BBHI: Alvaro Pascual-Leone (apleone@hsl.harvard.edu)(PI); bbhi@guttmann.com (AC); https://bbhi.cat/en
Harvard Aging Brain Study (HABS): Reisa Sperling; reisa@rics.bwh.harvard.edu (PI); habs@mgh.harvard.edu (AC); https://habs.mgh.harvard.edu
UKB: Rory Collins (rory.collins@ndph.ox.ac.uk) (PI); access@ukbiobank.ac.uk (AC); https://www.ukbiobank.ac.uk/
PREVENT-AD: Jennifer Tremblay; jennifer.tremblay-mercier@douglas.mcgill.ca (PI); https://openpreventad.loris.ca/contact/ (AC); https://prevent-alzheimer.net; https://openpreventad.loris.ca/
OASIS3: Pamela J. LaMontagne; pjlamontagne@wustl.edu (PI); Daniel Marcus; dmarcus@wustl.edu (PI); https://www.oasis-brains.org/#contact (AC); https://www.oasis-brains.org/
VETSA: William S. Kremen ( wkremen@ucsd.edu)(PI); https://www.vetsatwins.org/for-researchers/ (AC); https://www.vetsatwins.org/for-researchers/
PI = Principal Investigator. AC = Administrative contact.

# Research involving human participants, their data, or biological material

Policy information about studies with human participants or human data. See also policy information about sex, gender (identity/presentation), and sexual orientation and race, ethnicity and racism.

| | |
|---|---|
| Reporting on sex and gender | Sex (self-reported biological sex) was used as covariate in most models as well as used for sex-specific norms in normative modelling. Sex was not a measure of interest in any of the analyses. |
| Reporting on race, ethnicity, or other socially relevant groupings | Race, ethnicity, or other socially relevant groupings were not considered in this study. |
| Population characteristics | 13 different datasets were used so the specific exclusion/inclusion criteria varied slightly across studies. See Supplementary Information for more information. The common criteria was that individuals had to be >18 years of age, be followed longitudinally, having both 2 or more MRIs and cognitive assessments, and be cognitively healthy at any given observation and at baseline. |
| Recruitment | We used data from 13 different datasets and thus we inherited the different biases from the different datasets. Selection bias, and use of WEIRD samples were predominant. Attrition bias was also present: individuals that dropped-out from longitudinal studies are different than those that continue in several important aspects (e.g., are less healthy, have less cognitive function, are less educated, etc.). We do not believe these biases had a huge impact on the results; though it is possible that these biases, especially attrition bias, leads to somewhat attenuated results as individuals that drop-out are likely to have steeper MRI and cognitive change. |
| Ethics oversight | Regional committees for medical and health research ethics (REK) https://www.forskningsetikk.no/en/about-us/our-committees-and-commission/rek/ and the ethical approval #2010/3407 is called "Biologiske prediktorer for hukommelse - En oppfølgingsundersøkelse." The translation in english would be similar to "Biological predictors of memory. A longitudinal study". |

Note that full information on the approval of the study protocol must also be provided in the manuscript.

# Field-specific reporting

# Life sciences study design

All studies must disclose on these points even when the disclosure is negative.

| | |
|---|---|
| Sample size | Sample size was not estimated a priori. It represents a sample of convenience from legacy studies. We aimed for a large dataset (initial goal: >3k longitudinally-followed individuals) considering 1) low reliability of brain and cognitive change measures, which we estimated a priori (https://www.biorxiv.org/content/10.1101/2024.06.03.592804v1.), relatively suboptimal power for bootsrapping in GAMM models (https://journals.sagepub.com/doi/full/10.1177/25152459231207787) and relatively minor effects of 2 and 3 way-interactions in which we expected attenuation (but not reversal of effects) (e.g. DOI: 10.15200/winn.142559.90552). |
| Data exclusions | Data exclusion criteria varied slightly from dataset to datasets (see Supporting Information). In addition to cohort-specific inclusion and exclusion criteria, observations concurrent with cognitive impairment and Alzheimer's dementia were excluded. Individual with baseline age <20 or with severe neurological or psychiatric disorders were additionally excluded. Based on preprocessing requirements, MRI data from scanners with fewer than 25 observations were also excluded based on stability issues in the normative modeling-based harmonization. For computing slopes, individuals with less than 1.5 years of follow-up, either of memory function or brain structure were also excluded. For memory–brain pairing, individuals without at least partially overlapping follow-up intervals for brain structure and memory were excluded, as well as those in which one of the assessments started > 10 years before or ended > 10 years after the beginning or end of the MRI follow-up period. |
| Replication | We did not perform a replication with independent datasets as this would lead to suboptimal power. We carried control analyses for most analyses varying specific parameters of the statistical models (e.g., adding additional covariates, including random slopes, etc.) |
| Randomization | This is an observational study and randomization is not relevant. |
| Blinding | This is an observational study and blinding is not relevant. |

# Reporting for specific materials, systems and methods

We require information from authors about some types of materials, experimental systems and methods used in many studies. Here, indicate whether each material, system or method listed is relevant to your study. If you are not sure if a list item applies to your research, read the appropriate section before selecting a response.

## Materials & experimental systems

| n/a | Involved in the study | |
|---|---|---|
| ☒ | ☐ | Antibodies |
| ☒ | ☐ | Eukaryotic cell lines |
| ☒ | ☐ | Palaeontology and archaeology |
| ☒ | ☐ | Animals and other organisms |
| ☒ | ☐ | Clinical data |
| ☒ | ☐ | Dual use research of concern |
| ☒ | ☐ | Plants |

## Methods

| n/a | Involved in the study | |
|---|---|---|
| ☒ | ☐ | ChIP-seq |
| ☒ | ☐ | Flow cytometry |
| ☐ | ☒ | MRI-based neuroimaging |

# Plants

| | |
|---|---|
| Seed stocks | N/A |
| Novel plant genotypes | N/A |
| Authentication | N/A |

# Magnetic resonance imaging

## Experimental design

| | |
|---|---|
| Design type | N/A |
| Design specifications | N/A |

Behavioral performance measures | N/A

## Acquisition

Imaging type(s) | T1-weighted structural images

Field strength | 1.5, 3.0T (several scans)

Sequence & imaging parameters | MPRAGE and 3D FSPGR sequences. Imaging parameters vary from scan to scan (>50 scanners; see Supplementary Table 9).

Area of acquisition | Brain scans

Diffusion MRI ☐ Used ☒ Not used

## Preprocessing

Preprocessing software | longitudinal FreeSurfer v.7.1.0 stream

Normalization | surface-based normalisation for cortical stream / mri_robust_register (computes symmetric robust registration via talairach.xfm)

Normalization template | MNI305 / fsaverage

Noise and artifact removal | N/A

Volume censoring | N/A

## Statistical modeling & inference

Model type and settings | Brain regions (cortical thickness (Destrieux atlas) and subcortical volume (aseg atlas)) were harmonized using a normative modelling framework with the PCNtoolkit (0.30.post2), in Python3 environment (version 3.9.5). PCNtoolkit uses a Hierarchical Bayesian Regression (HBR) technique and pretrained models from 82 different datasets, including the UKB. New sites can be added to the model using a held-out calibration dataset. We calibrated data iteratively not to loose longitudinal individuals. ICC was used to estimate the adequacy and reliability of this method. Individual change was computed using linear models per individual

Effect(s) tested | Relationship between brain change and memory change were assessed with generalized additive mixed models (GAMMs). Analyses involving APOE status and age also used GAMM models. The association between APOE status and brain/cognitive change was assessed with linear mixed effects models (lme)

Analyses were carried out as a response to reviewers. These include: a) comparison between harmonization methods (GAMM vs normative modelling), b) Effect of education on brain decline, cognitive decline, and the association between brain change and cognitive change; c) Reran APOE status analyses using individuals aged 60 years or more

Specify type of analysis: ☐ Whole brain ☒ ROI-based ☐ Both

Anatomical location(s) | Cortical thickness: Destrieux atlas; Subcortical volume: aseg atlas as implemented in the longitudinal FreeSurfer stream.

Statistic type for inference (See Eklund et al. 2016) | N/A

Correction | multiple comparisons correction (FDR) and bootstrapping to obtain p-values with a proper false-positive ratio.

## Models & analysis

| n/a | Involved in the study |
|---|---|
| ☒ | ☐ Functional and/or effective connectivity |
| ☒ | ☐ Graph analysis |
| ☐ | ☒ Multivariate modeling or predictive analysis |

Multivariate modeling and predictive analysis | A secondary analyses involved a principal component analyses (PCA) and a monte-carlo based consensus clustering algorithm (M3)
